# Impact of body mass index on opioid consumption in lumbar spine fusion surgery

**DOI:** 10.1016/j.xnsj.2021.100060

**Published:** 2021-04-08

**Authors:** Taryn E. LeRoy, Andrew S. Moon, Marissa Gedman, Jessica P. Aidlen, Ashley Rogerson

**Affiliations:** a Department of Orthopaedic Surgery, Tufts Medical Center, 800 Washington Street, Boston, MA 02111, United States; b Tufts University School of Medicine, 145 Harrison Avenue, Boston, MA 02111, United States; c Department of Orthopaedic Surgery, Newton Wellesley Hospital, 2014 Washington Street, Newton, MA 02462, United States

**Keywords:** Lumbar fusion, Interbody fusion, Obesity, Opioid consumption, Opioids, Outcomes

## Abstract

**Background:**

in the United States from 1999 to 2000 through 2017–2018, the prevalence of obesity increased from 30.5 to 42.4%, while the prevalence of severe obesity nearly doubled. In lumbar spine surgery, obesity is associated with increased complications, worse perioperative outcomes, and higher costs. The purpose of this study was to examine the association between body mass index (BMI) and opioid consumption in patients undergoing lumbar spine fusion surgery. We hypothesized that obese patients would require more opioids postoperatively.

**Methods:**

retrospective review of 306 patients who underwent one- or two-level posterior lumbar interbody fusion surgery between 2016 and 2020. Patients were stratified by BMI as follows: normal weight (18.5–24.9 kg/m2), overweight (25.0–29.9 kg/m2), obese I (30.0–34.9 kg/m2), and obese II–III (≥ 35.0 kg/m2). Patient demographics and preoperative characteristics were compared across BMI cohorts using one-way ANOVA and chi-square analysis. Patients with prior history of opioid use were excluded. Primary outcome measure was postoperative opioid consumption. Secondary outcomes included operative time, length of stay (LOS), discharge destination, and 30-day re-encounter rates. Outcomes were analyzed using multivariable linear regression adjusted for potential confounders.

**Results:**

of 306 total patients, 17.3% were normal weight, 39.9% were overweight, 25.5% were obese I, and 17.3% were obese II–III. Obesity was associated with longer operative times and length of stay (*p* < 0.001, *p* = 0.024). For opioid naïve patients, there was no difference in-house opioid consumption when adjusted for kilograms of body mass and LOS (*p* = 0.083). Classes II–III patients were prescribed more than twice the number of postoperative opioids (*p* < 0.001) and were on opioids for a longer time postoperatively (*p* = 0.019).

**Conclusion:**

obesity is associated with longer operative times, longer LOS, and increased consumption of postoperative opioids. This should be considered when counseling patients preoperatively prior to lumbar spine fusion procedures.

## Introduction

Obesity is a public health crisis in the United States, with nearly forty percent of American adults over the age of 20 considered obese [1]. This has doubled since 2000 [2]. Excess weight and obesity have been linked to significant medical comorbidities including diabetes, hypertension, dyslipidemia, coronary artery disease, respiratory problems, sleep apnea, and some cancers [3,4]. Regarding spine health, obesity has been associated with a higher prevalence of chronic low back pain and intervertebral disc degeneration [[5], [6], [7], [8]]. Studies have also reported a higher rate of perioperative complications, less favorable outcomes, and higher costs in obese patients [7,[9], [10], [11].

Prior research has shown that pain and obesity are significantly associated with one another [[12], [13], [14], [15], [16]], with both structural and chemical mechanisms underlying this relationship [12]. Morbidly obese people are four times more likely to have a pain complaint compared to those who are not obese [13], and the prevalence of low back pain has been shown to have a direct linear correlation with BMI [14]. Both obesity and back pain negatively impact health related quality of life and account for a large proportion of U.S. health care expenditures [17,18].

With the association between pain and obesity, the question arises as to whether obesity is also linked to increased opioid consumption. Chronic opioid use both before and after lumbar spine surgery is associated with higher healthcare utilization and worse postoperative outcomes [[19], [20], [21]]. To our knowledge, there is no literature to date examining the association between opioid consumption after lumbar spine fusion and BMI. Better understanding of the association between BMI and opioid consumption will allow opportunities for more effective patient counseling regarding postoperative pain control. We sought to examine the association between BMI and postoperative opioid consumption in opioid naïve patients undergoing one- or two-level posterior lumbar interbody fusion surgery.

## Material and methods

### Patient population

Study approval was obtained through the hospital's institutional review board. A retrospective review was performed evaluating all patients who had undergone 1or 2 level posterior lumbar interbody fusion surgery during the study period of April 1, 2016 to March 31, 2020. All procedures were performed by three surgeons at a single institution. Indications for surgery included spinal stenosis, lumbar spondylosis and spondylolisthesis, and degenerative disc disease. Exclusion criteria included trauma, malignancy, infection, more than two fusion levels, and revision fusion.

### Data collection

Patient and surgical data were collected by review of the institutional electronic medical record. Patient demographics included age, sex, American Society of Anesthesiologists (ASA) score, body mass index (BMI), depression, preoperative opioid use, and tobacco use. ASA score was obtained from the anesthesiologist record from the day of surgery. Patient's height and weight were reported on day of surgery per hospital/anesthesia protocol. This was used to calculate BMI.

Patients were grouped by BMI category as follows: normal weight (18.5–24.9 kg/m^2^), overweight (25.0–29.9 kg/m^2^), obese I (30.0–34.9 kg/m^2^), and obese II–III (>/= 35.0 kg/m^2^) [2]. History of depression was obtained from the patient's past medical history.

Preoperative opioid use status was determined based on prior opioid prescriptions tracked in the electronic medical record. A patient was considered to be opioid naïve if they had not taken opioids within 6 months of surgery. Tobacco use was self-reported and listed in the social history in the patient's medical record.

Operative information included the number of operative levels, procedure type (open vs. minimally invasive), operative time, intraoperative estimated blood loss (EBL), and intraoperative complications. Operative time was defined as time from incision to time of completion of closure. Minimally invasive transforaminal lumbar interbody fusion (MIS-TLIF) via the paramedian approach was considered minimally invasive and posterior midline incision was considered open.

Hospitalization data included in-house and post-discharge opioid consumption, length of stay, discharge location, and 30-day readmission or presentation to a medical center. Length of stay was defined as the number of days spent in the hospital from the day of surgery to the day of discharge. Discharge destination was recorded as either discharge to home or to an interim rehabilitation facility. Opioid naïve patients were compared across BMI groups.

### Opioid consumption

In-house opioid consumption was calculated by totaling all opioids administered during the patient's hospitalization converted to morphine equivalent doses (MED). All patients received a standardized postoperative pain regimen while in-house. This regimen includes Acetaminophen 1000 mg three times daily, a muscle relaxant, an oral opioid as needed (prn) for pain and an IV opioid for breakthrough pain. Oral opioid options include Tramadol 25–50 mg every six hours prn, Oxycodone 5–15 mg every four hours prn, and Dilaudid 2–6 mg every four hours prn. The muscle relaxant prescribed is Tizanidine 2–4 mg every eight hours prn and the breakthrough IV medication is hydromorphone (0.5–1.0 mg every two hours prn).

Post-discharge opioid consumption was determined based on the number of prescriptions a patient received postoperatively. Date of the last prescription was used to determine length of time on opioids after surgery. Opioid prescription data included the type of opioid and number of pills prescribed. The total number of prescriptions and number of pills prescribed were calculated and converted to MED. Each patient's weight at time of surgery was used to determine MED consumption per unit weight in MED/kg.

### Statistical analysis

All statistical analyses were performed using SPSS 26.0 (IBMCorp). Patient demographics and perioperative characteristics were compared across the BMI cohorts using one-way ANOVA and chi-square analysis for continuous and categorical variables, respectively. Multivariable linear regression was used to determine presence of an association between BMI category and opioid utilization after surgery. Multivariable analyses were adjusted for age, sex, ASA level, history of depression, and smoking status. Statistical significance was set at *p* < 0.05. Patients with missing data points were excluded from statistical analysis for that particular variable.

## Results

Of a total of 306 patients who underwent 1 or 2 level posterior lumbar interbody fusion surgery during the study period, 17.3% (*N* = 53) were normal weight, 39.9% (*N* = 122) were overweight, 25.5% (*N* = 78) were obese I, and 17.3% (*N* = 53) were obese II–III. There was no difference in age, sex, preoperative opioid use, depression, procedure type, discharge destination or 30-day re-encounter rates (Table 1). There was a statistically significant difference in ASA (*p* = 0.001), tobacco use (*p* = 0.047), operative time (*p* < 0.001), and length of stay (3.02 days, *p* = 0.019). (Table 1).

**Table 1:** Demographic data compared across levels of obesity.

BMI Classification	Normal	Overweight	Obese I	Obese II–III	*P-Value*
Total patients, *N* = 306*	53 (17.3)	122 (39.9)	78 (25.5)	53 (17.3)	–
Age	61.49 ± 13.32	59.96 ± 11.78	60.69 ± 11.72	57.53 ± 11.47	0.345
Sex (F)	34 (54)	49 (40.2)	37 (47.4)	26 (49)	0.122
ASA ≥3*	8 (13.5)	26 (21.3)	27 (34.6)	24 (45.3)	0.001
Pre-op opioid use (Y)*	18 (34)	24 (20)	22 (29)	18 (34)	0.111
Depression (Y)*	11 (20.7)	21 (17.2)	15 (19.2)	10 (18.8)	0.867
Tobacco (current smoker)*	7 (13.2)	14 (11.4)	1 (0.01)	6 (11.3)	0.047
Procedure (“mini”)*	12 (22.6)	40 (32.7)	20 (25.6)	12 (22.6)	0.376
EBL (ml)	144.91 ± 146.12	175.04 ± 152.82	226.28 ± 256.90	217.83 ± 172.30	0.049
OR time (min)	138.87 ± 35.72	166.29 ± 48.41	175.69 ± 50.85	181.25 ± 49.65	<0.001
Length of stay (days)	2.11 ± 1.17	2.45 ± 1.34	2.56 ± 1.73	2.98 ± 1.62	0.024
Rehab (Y)*	6 (11.3)	12 (9.8)	9 (11.5)	12 (22.6)	0.122
30-day re-encounter*	5 (9.4)	12 (9.8)	5 (6.4)	5 (9.4)	0.857

⁎ The values are given as the number of patients, with percentage of the total in parentheses.

After excluding patients with a history of opioid use (*N* = 84), opioid naïve patients were compared across BMI groups. There was no difference in age, depression, EBL, length of stay, discharge destination or 30-day re-encounter rates (Table 2). There was a statistically significant difference in sex (*p* = 0.021), ASA (*p* = 0.027), tobacco use (*p* = 0.043), and operative time, with longer operative times in obese II–III patients (*p* < 0.001) (Table 2).

**Table 2:** Demographic data compared across levels of obesity for opioid naïve patients only+.

BMI Classification	Normal	Overweight	Obese I	Obese II–III	*P-Value*
Total patients, *N* = 224*	34(15.2)	97 (43.3)	55 (24.6)	35 (15.6)	–
Age	63.4 ± 13.92	60.27 ± 11.19	61.04 ± 11.73	58.97 ± 12.32	0.444
Sex (F)	24 (71)	40 (41.2)	23 (41.8)	18 (22.9)	0.021
ASA ≥3*	6 (17.6)	20 (20.6)	18 (32.7)	15(42.9)	0.027
Depression (Y)*	4 (12.5)	10 (14.7)	9 (19)	4 (12)	0.868
Tobacco (current smoker)*	5 (10)	9 (9.5)	0 (0)	5 (8.8)	0.043
EBL (ml)	156.57 ± 152.44	160.26 ± 132.95	224.20 ± 254.35	196.29 ± 129.98	0.138
OR time (min)	142.14 ± 38.93	164.54 ± 49.49	173.94 ± 49.63	193.74 ± 45.07	<0.001
Length of stay (days)	1.86 ± 0.88	2.40 ± 1.38	2.57 ± 1.93	2.63 ± 1.21	0.094
Rehab (Y)	4 (10)	8 (9.5)	5 (11.3)	5 (8.8)	0.742
30-dayre-encounter*	3 (12.5)	10 (10.5)	3 (4)	5 (15)	0.539

⁎ The values are given as the number of patients, with percentage of the total in parentheses.

+ *A* patient is opioid naïve if they have not taken opioids within 6 months of surgery.

After excluding preoperative opioid users, there was no difference in-house opioid consumption across BMI groups when controlled for length of stay (*p* = 0.085) (Table 3). Furthermore, when adjusting opioids to MED per kg of patient's body weight, there was no significant difference in-house opioid consumption (*p* = 0.083) (Table 3).

**Table 3:** Opioid use by level of obesity for opioid naïve patients only+.

BMI Classification	Normal	Overweight	Obese I	Obese II–III	*P-Value*
Total Patients, *N* = 224*	40 (18)	95 (42.7)	53 (23.8)	34 (15.3)	–
In-house opioids (MED)	116.92 ± 96.17	190.83 ± 133.59	188.68 ± 162.81	217.85 ± 156.70	0.018
In-house opioids (MED/day)	62.57 ± 42.47	82.02 ± 46.90	74.78 ± 39.11	89.69 ± 64.50	0.085
In-house opioids (MED/kg/day)	0.93 ± 0.59	1.03 ± 0.58	0.82 ± 0.44	0.82 ± 0.62	0.083
Total post-discharge opioids (MED)	1036.29 ± 1289.66	1272.19 ± 1148.30	1352.65 ± 1592.89	3013.68 ± 4740.94	<0.001
Total post-discharge opioids (MED/kg)	15.34 ± 18.09	16.02 ± 13.70	14.14 ± 15.64	27.29 ± 43.13	0.034
Total # days on opioids post-discharge	17.06 ± 32.04	24.1 ± 39.0	36.09 ± 73.85	63.32 ± 124.15	0.019

⁎ The values are given as the number of patients, with percentage of the total in parentheses.

+ *A* patient is opioid naïve if they have not taken opioids within 6 months of surgery.

There was a significant difference in postoperative opioid consumption with obese classes II–III prescribed more than double the number of opioids compared to patients with normal BMI (3013.68 MED ± 4740.94 vs. 1036.29 MED ± 1289.66; *p* < 0.001) (Table 3). When evaluating post-discharge opioid consumption based on patient's body mass (MED/kg), patients in obese II–III continued to consume more opioids postoperatively (*p* = 0.034). Obese classes II–III patients remained on opioids for a significantly longer time postoperatively (*p* = 0.019). In the multivariable regression analysis, the only BMI group associated with a significant increase in postoperative opioid consumption was obese classes II–III (*p* = 0.013) (Table 4).

**Table 4:** Multiple regression results for BMI class x postoperative MED/kg.

BMI Classification*	Overweight	Obese I	Obese II–III
B	132.19	272.46	1362.50
Std Error	436.58	501.26	545.39
*P-Value*+	0.762	0.587	0.013

⁎ BMI classes were compared against the ‘Normal’ class which was used as the constant.

+ Multiple regression was run holding age, sex, ASA, smoking status, levels fused and surgical technique constant.

## Discussion

This study examined the association between two major public health concerns: obesity and the opioid epidemic. Our data shows that patients who are in obese classes II–III (BMI ≥ 35.0) were prescribed more than double the number of opioids postoperatively and were on opioids for a significantly longer time postoperatively. This suggests that obesity increases the need for postoperative opioids after lumbar spine interbody fusion surgery.

The use of prescription opioids in the U.S. has increased fourfold between 1997 and 2010. This increase has been associated with increased misuse of these medications, diminished quality of life, increased pain intensity and pain-related disability, and increased morbidity and mortality [[21], [22], [23], [24]]. Although opioids are useful in the acute postoperative period, prescribing opioids after orthopedic surgery has been linked to increased conversion of opioid naïve patients to opioid dependent patients after surgery [20,25]. Patients with chronic pain managed with long-term opioid therapy who undergo surgery are also at greater risk for complications and poor postoperative outcomes [21,23,26].

Obesity in lumbar spine fusion surgery has been shown to be associated with worse postoperative outcomes, increased complications, and higher costs [7,11,27,28]. However, there is a paucity of information examining postoperative opioid use in obese patients after lumbar spine fusion surgery. A study by Narain et al. looked at inpatient opioid consumption across BMI groups in patients undergoing anterior cervical discectomy and fusion and found no significant difference in morphine equivalent consumption during a patient's hospital admission [29]. Similarly, we found no difference in in-house opioid consumption across BMI groups. This is likely due to the standardized postoperative pain order set with scheduled medications including acetaminophen as well as prn medications including an oral opioid, tizanidine, and IV medication for breakthrough pain. The similar intake of opioids may in part be due to the fact that nursing and other clinical staff evaluate the patients throughout their hospitalization and assess their pain level on a pain scale during clinical assessments. We expected there would not be a difference in inpatient opioid consumption; however, once patients are independently managing their pain after discharge, there is a significant difference in opioid requirements.

It is well established that obesity is independently linked to longer operative times [10,30,31]. Patients with higher BMI tend to have a thicker layer of subcutaneous tissue, necessitating a larger dissection, more retraction, and increased time for dissection. This leads to increased operative time. A meta-analysis by Jiang et al. found that obese patients were fourteen times more likely to have longer operative times compared to non-obese patients [10]. Goyal et al. examined 14 studies with over 6000 patients and found that obesity is associated with significantly higher operative times, both for MIS procedures and open spine surgeries [31]. Our findings are consistent with the literature in that obese patients in classes II–III had significantly longer operative times.

The association between obesity and prolonged length of stay is conflicting in the literature. Our data shows that obesity was associated with a prolonged length of stay. Similarly, Seicean et al. examined nearly 50,000 patients undergoing spine surgery and found that patients in both obese classes II and III had increased odds of prolonged length of stay [32]. However, there are other studies that have found no difference in length of hospitalization between obese and non-obese patients [31,33,34].

Higher BMI was not associated with increased 30-day re-encounter rates after surgery in our study. The association between obesity and postoperative outcomes and complications is unclear. Numerous studies have shown increased postoperative complications with increasing BMI [11,35,36]. Complications include wound infection, cerebrospinal fluid leak, deep venous thrombosis, cardiac events, pneumonia, prolonged intubation, readmission, and return to the operating room. Cao et al. found significant differences with prolonged operating time, blood loss, surgical site infection, and nerve injury; however, there was no difference in deep venous thrombosis, dural tear, revision, and mortality [30]. Other studies have found that BMI is not associated with an increased incidence of minor or major complications [27,33,37]. In this study, nine percent of patients presented to the ED or were readmitted within 30 days for various complications ranging from wound infection to urinary tract infection; however, there were no significant associations with BMI.

It is imperative that surgeons counsel overweight and obese patients on the importance of weight loss prior to spine surgery. Obesity strains the axial skeleton and increases the mechanical load imparted on the spine [38]. It has been shown that obesity contributes to low back pain, sciatica, disc degeneration, and increases the risk of developing operative pathology [39]. Attaining a healthy weight may reduce or eliminate a patient's back pain. Although spine surgery in obese patients may lead to clinical improvements in both pain and disability, obese patients may be at higher risk for complications. It is imperative that surgeons counsel obese patients on both the risks and benefits of surgery in the setting of their obesity. It is also important to discuss that weight loss may not only mitigate complications but may actually lead to a patient having less back pain, shorter operative time, and improved clinical outcomes after surgery.

We recognize the limitations of the study. First, while we were able to quantify the number of pills patients were prescribed postoperatively, we understand that writing a prescription does not mean a patient consumed the tablets. A patient could choose to not fill the prescription or fill the prescription, but not consume the entire prescription. However, this is a limitation across all patients, regardless of BMI. Presumably patients on chronic opioids will remain on opioids after surgery, therefore we were unable to quantify post-discharge opioid consumption in this population. Another limitation is that three surgical techniques for interbody fusion were utilized by three surgeons, thereby this could be considered a heterogeneous population. However, all patients underwent either 1 or 2 level fusion and no procedure was a revision fusion surgery.

To our knowledge, this is the first study that focuses on postoperative opioid consumption after lumbar spine fusion surgery across BMI groups. These findings show that obesity is associated with increased opioid consumption postoperatively in both chronic opioid users and opioid naïve patients. Few studies have specifically analyzed the effect of obesity on postoperative opioid usage. We hope these findings stimulate interest in further evaluating prescribing patterns based on obesity after lumbar spine surgery and help surgeons counsel patients about the risk of prolong opioid use in obese patients.

## Conclusions

As the prevalence of obesity increases, there will likely be more patients with low back pain and degenerative disc disease, leading to a higher percentage of patients needing surgical treatment for lumbar spine pathology. It is imperative that we counsel patients on the importance of weight loss as this may mitigate the need for spine surgery. Furthermore, it is important to educate patients on the opioid epidemic, postoperative pain, and opioid consumption. Surgeons should counsel obese patients that they are at higher risk for increased opioid consumption and may require more of a multimodal pain regimen postoperatively to limit this risk.

## Funding disclosure statement

There was no financial support for this study.

## Declarations of Competing Interests

The authors declare that they have no known competing financial interests or personal relationships that could have appeared to influence the work reported in this paper.
