# Supplementary material for: Impact of body mass index on opioid consumption in lumbar spine fusion surgery
Source: N Am Spine Soc J. 2021 Apr 8;6:100060. doi: 10.1016/j.xnsj.2021.100060 (PMC8820039; doi:10.1016/j.xnsj.2021.100060)
Supplement: Supplementary file 1 [file mmc1.docx]

# THE SPINE JOURNAL

**A Multidisciplinary Journal of Spinal Disorders**

***Official Journal of the North American Spine Society***

**FDA APPROVAL STATUS**

Article Title:

Impact of body mass index on opioid consumption in lumbar spine fusion surgery

Manuscript reference #:

**FDA Approval Status**

If a device or drug requiring FDA approval is identified as an important component of your article, you must indicate the FDA status for use as it will be discussed. Please list the name of the device(s) and drug(s) requiring FDA approval and check the appropriate status for use as it is discussed in the article.

**J**


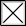
My manuscript does not discuss any drugs or devices requiring FDA approval.

1. Device/Drug


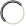

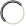

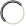

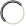
Not Applicable Not approved for this indication Approved Investigational

1. Device/Drug


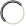

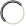

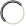

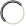
Not Applicable Not approved for this indication Approved Investigational

1. Device/Drug


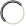

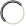

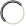

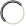
Not Applicable Not approved for this indication Approved Investigational

1. Device/Drug


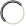

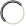

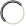

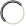
Not Applicable Not approved for this indication Approved Investigational

1. Device/Drug


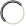

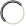

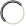

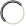
Not Applicable Not approved for this indication Approved Investigational

Corresponding Author Name: Date:

Ashley Rogerson, MD

Sep 10, 2020
